# Flavoring Production in Kamut®, Quinoa and Wheat Doughs Fermented by *Lactobacillus paracasei, Lactobacillus plantarum*, and *Lactobacillus brevis*: A SPME-GC/MS Study

**DOI:** 10.3389/fmicb.2018.00429

**Published:** 2018-03-09

**Authors:** Tiziana Di Renzo, Anna Reale, Floriana Boscaino, Maria C. Messia

**Affiliations:** ^1^Institute of Food Science, National Research Council, ISA-CNR, Avellino, Italy; ^2^Department of Agricultural, Environmental and Food Sciences, DiAAA, University of Molise, Campobasso, Italy

**Keywords:** flavor, quinoa, Kamut®, *Lactobacillus paracasei*, SPME-GC/MS, lactic acid bacteria, sourdough

## Abstract

This study identified the odor-active compounds and the qualitative characteristics of doughs from “ancient” grains flours fermented by lactic acid bacteria. For this purpose doughs made with quinoa and Kamut® flours have been produced and inoculated with strains belonging to the species *Lactobacillus paracasei, Lactobacillus plantarum* and *Lactobacillus brevis* and compared with fermented doughs made from 100% wheat flour. The quality of the doughs was determined by assessment of pH, total titratable acidity, lactic acid bacteria growth and flavor compounds. The results showed that lactic acid bacteria used were able to grow in the different substrates reaching more than 9.0 log CFU/g after 24 h fermentation, although the best microbial growth was recorded in the doughs made with quinoa flour fermented with *Lactobacillus paracasei* I1. Good acidification and heterogeneous aromatic profile were recognized in all the doughs even if the volatile composition mainly derived from microbial specie. Among all the used strains, mostly *Lactobacillus paracasei* I1 positively contributed to the aromatic profile of the doughs, independently from flour type, producing the highest amount of different ketones such as, diacetyl, acetoin, 2,6-dimethyl-4-heptanone, 5-methyl-3-hexanone, 4-methyl-3-penten-2-one, volatile compounds highly appreciated in the bakery products for their buttery, fatty and fruity notes. So, the positive characteristic of *Lactobacillus paracasei* I1 to enhance the production of desired volatile compounds could make it suitable as adjunct culture starter in the bakery industry. Many differences in volatile organic compounds derived also by the type of flour used. Quinoa fermented doughs were characterized for specific nutty, roasted, acid and buttery tones derived from pyrazines, ketones and acid compounds whereas Kamut® fermented doughs were characterized for fruity, rose, green and sweet tones derived from aldehydes and ketones production. So, the use of quinoa and Kamut® flours opportunely fermented, as partial or complete substitution of wheat flour, may be interesting for producing more balanced bakery products with respect to nutritional aspects and to unique aromatic profile. Furthermore, the supplementation of these flours, rich in protein content and free amino acids, could represent an optimal substrate to enhance the growth of lactic acid bacteria used as starter culture in leavened bakery products.

## Introduction

Wheat (*Triticum aestivum*) is the cereal most commonly used to produce numerous bakery products but the evolution of food habits and the increased consumer desire for a healthy lifestyle, determined a notable renovation of the baked products market resulting in demands for breads and bakery products with highly nutritional and functional properties. One of the possibilities explored by bakers to adapt their products to the market demands is the total or partial replacement of wheat flour with alternative flours with nutritional and healthy distinctive features (Balestra et al., 2015). In the recent years, great interest is enjoying the use of flours obtained from “ancient” grains, especially cereals as Kamut® khorasan wheat, emmer, barley and spelt, and pseudocereals, such as sorghum, teff, millet, amaranth, and quinoa for the formulation of new bakery products having improved nutritional characteristics and distinguishing flavor (Bhargava et al., 2006; Zannini et al., 2009; Jekle et al., 2010; Vega-Gálvez et al., 2010; Angioloni and Collar, 2011; Mariotti et al., 2014). Due to their higher content of certain components, such as, essential aminoacids, dietary fiber, vitamins and minerals, but also other bioactive molecules such as, omega-3 fatty acids, prebiotic oligosaccharides, phytosterols, polyphenols (Bordoni et al., 2017), these grains are considered suitable for the healthy food production and special dietary uses (Di Cagno et al., 2004; Sterr et al., 2009).

In this respect, among the old grains varieties, khorasan wheat (Kamut®) has emerged as one of the most important for its beneficial effects on human health. It is an ancestor variety of grain (*Triticum turgidum* var. *turanicum* commonly called khorasan wheat), that is a registered trademark of Kamut® International, Ltd. (Big Sandy, MT) and Kamut® Enterprises of Europe (Oudenaarde, Belgium), that guarantees certain attributes, mainly a protein content of 12–18%. It has been shown that Kamut® khorasan bread protects from oxidative stress and inflammatory status to a greater extent than that afforded by whole-grain durum wheat (Benedetti et al., 2012; Sofi et al., 2013). Therefore this type of grain can be used successfully to obtain bakery products with an increased nutritional and functional value (Valli et al., 2016).

Other than khorasan Kamut®, recently also the use of pseudocereals, in particular quinoa, is strongly increased especially due to its abundance in regard to bioactive compounds and techno-functional substances and to its lack of allergenic proteins (Elgeti et al., 2014). Quinoa (*Chenopodium quinoa* Willd.), a pseudo-cereal native to the high altitude soils of the Andean region of South America (Matiacevich et al., 2006), is now widely used for its outstanding nutritional qualities (Repo-Carrasco et al., 2003; Ruiz et al., 2014) and for the absence of gluten (Alvarez-Jubete et al., 2009).

The partial or complete substitution of wheat flour with flours from “ancient” grains could add nutritional value to the final products, provided that the substituting flours not negatively affect the sensorial and technological characteristics of finished bakery products. Furthermore, considering that the manufacture of different leavened baked products such as, breads, cakes and crackers are made by use of fermented sourdoughs, the activities of lactic acid bacteria involved could have a critical impact on the final quality of alternative cereal-based products.

Lactic acid bacteria, in fact, play an important role during sourdough fermentation, especially for the development of flavor components (Damiani et al., 1996; Corsetti and Settanni, 2007; Reale et al., 2016) other than nutritional and rheological quality of the finished baked products (Katina et al., 2006a; Poutanen et al., 2009; Jekle et al., 2010; Dallagnol et al., 2013; Gobbetti et al., 2014). Besides the baking process, which mainly influences the typical aroma of the bread crust, the most important step in the development of crumb flavor is dough fermentation (Hansen and Schieberle, 2005). Some of the compounds occur in bread have been shown to be related to the concentrations in the corresponding sourdoughs. As an example, the contents of methylpropanol, 2- and 3-methylbutanol, ethyl acetate and ethyl lactate in three bakery sourdoughs were clearly related to the amounts of these compounds in the sourdoughs (Hansen et al., 1989).

The choice of the starter cultures that prevail and compete with contaminants in sourdough fermentation may have a strong impact on the final sensorial characteristics of the products and may direct toward the production of specific aromas. The combined use of specific microbial starter and alternative flours could allow to produce more balanced foods with respect to distinct nutritional properties and to aromatic features than conventional wheat bakery products responding to new market requirements.

On these bases, the present work was planned with the aim to evaluate the adaptability of different species of lactic acid bacteria (*Lactobacillus paracasei, Lactobacillus plantarum* and *Lactobacillus brevis*) to sourdough obtained using conventional (wheat) and non-conventional flours (quinoa and Kamut®) alone or in combination and the influence of fermentation activities on the volatile profile of the final fermented doughs.

## Materials and methods

### Chemical composition of flours

Flour samples of wheat, quinoa and Kamut® were purchased in local store and were subjected to analysis of moisture (ICC method 109/1) (ICC, 1995), dietary fiber (AACC Method 32.05) (AACC, 2000), ash content (ICC method 104/1) (ICC, 1995), total protein content (ICC method 105/2) (ICC, 1995) and fat (AACC method 30-20) (AACC, 2000). Carbohydrates content was calculated by difference. The amino acid content was determined using the Dionex system (Dionex Corporation, Sunnyvale, CA, USA), composed of a gradient pump (mod GP50) with on-line degasser and an electrochemical detector (model ED40). The instrument control, data collection and total quantification were managed using Peak Net chromatography software (Dionex). Separation was performed with an Aminopac PA10 analytical column (250 × 2 mm, 8.5 μm particle size). The quantitative determination was carried out as described by Messia et al. (2005).

Data reported for all parameters were the average values of three different aliquots of each sample. All results are expressed as g/100 g dry weight (d.w.).

### Strains and culture conditions

Two homo-fermentative strains of lactic acid bacteria (LAB), *Lactobacillus paracasei* I1 (pa) and *Lactobacillus plantarum* M4 (pl) and one hetero-fermentative strain of *Lactobacillus brevis* T4 (br) (Reale et al., 2011), previously isolated from traditional ripe wheat sourdoughs, were used in this study. The strains were maintained as frozen stock at −80°C in reconstituted 11% (w/v) skim milk (Oxoid, Milan, Italy) containing 0.1% (w/v) of ascorbic acid in the culture collection of the Institute of Food Sciences of the National Research Council of Italy and were routinely propagated in MRS broth (Oxoid, Milan, Italy), pH 6.8, for 16 h at 30°C.

### Sourdough preparation

Commercially available wheat, quinoa and Kamut® flours were used for the realization of four types of doughs: W, 100% wheat flour; Q, 100% quinoa flour; K, 100% Kamut® flour; WQK, 25% wheat, 25% quinoa and 50% Kamut® flour. Each dough was prepared in sterile beaker mixing 100 g of flour and 50 mL of sterilized water, adding microbial starter when necessary. To each dough 1 g/L cycloheximide was added to inhibit indigenous yeasts as reported in Zotta et al. (2006).

Lactic acid bacteria strains were used as starter (~7, 7.5 log CFU/g) in each dough. Cells were harvested by centrifugation (12,000 g, 10 min, 4°C), washed twice, and resuspended in 0.85% (w/v) NaCl to obtain the desired final concentration. A dough un-inoculated was used as control. Therefore, 12 different types of dough were prepared (Wc, Qc, Kc, WQKc, control samples without microbial starter; Wpa, Qpa, Kpa, WQKpa, doughs inoculated with *L. paracasei* I1; Wpl, Qpl, Kpl, WQKpl, doughs inoculated with *L. plantarum* M4; Wbr, Qbr, Kbr, WQKbr, doughs inoculated with *L. brevis* T4) and fermented for 48 h at 30°C. Two biological replicates of each dough formulation were performed on separated days. At time zero and after 24 and 48 h of fermentation, pH, Total Titratable Acidity values (TTA), LAB counts and the production of volatile organic compounds (VOCs; by SPME-GC/MS), were evaluated and described below.

### pH and total titratable acidity assessment

The pH was determined with a pHmeter Medidor PH Basic 20 (CRISON, Spain). Total titratable acidity (TTA) was measured on 10 g of dough samples after homogenization in 90 mL of distilled water for 2 min in a Stomacher laboratory blender (BAG MIXER 400, Interscience, France). TTA values were expressed as the amount (mL) of 0.1 N NaOH necessary to achieve pH 8.3.

### Lactic acid bacteria count

For lactic acid bacteria count, 10 g of each dough was aseptically transferred into a sterile stomacher bag and diluted with 90 mL of physiological solution (9 g/L NaCl). After 1 min of agitation in a Stomacher, the samples were serially diluted and plated in duplicate. Lactic acid bacteria were counted on MRS (Oxoid, Milan, Italy) agar medium supplemented with 4 mg/100 mL cycloheximide (SIGMA Aldrich, Germany) after incubation at 30°C for 72 h in anaerobic conditions (Gas Pack AnaeroGenTM, Oxoid).

### Characterization of volatile organic compounds (VOCs)

The volatile fraction of samples was analyzed by headspace sampling, using the solid phase micro-extraction technique (SPME) according to Reale et al. (2016). In detail, for each SPME analysis, 2 g of samples were placed into a 20 mL headspace vial, and added 5 μL of 4-methyl-2-pentanol (internal standard, 100 mg/L standard solution). The vial was placed in a thermostatic block (40°C) on a stirrer and the fiber was inserted and maintained in the sample head space for 30 min, than it was removed and immediately inserted into the GC/MS injector for the desorption of compounds. For the analyses, a silica fiber, coated with 85 mm of CarboxenePolydimethylsiloxane (Carboxen/PDMS) was used (Supelco, Bellefonte, PA, USA).

### Gas chromatography/mass spectrometry (GC/MS) analysis

VOCs evaluation was carried out as described by Reale et al. (2016). In detail an Agilent Technologies (Agilent Technologies, USA) 7890 A gas chromatograph coupled to an Agilent Technologies 5975 mass spectrometer equipped with a 30 mx 0.25 mm ID, film thickness 0.25 μm capillary column (HP-INNOWAX, Agilent Technologies, USA) was used. Gas carrier was Helium (flow 1.5 mL/min) and SPME injections were splitless (straight glass line, 0.75 mmI.D.) at 240°C for 20 min during which time thermal desorption of analytes from the fiber occurred. The oven parameters were as follows: initial temperature was 40°C held for 3 min, followed by an increase to 240°C at a rate of 5°C/min, then held for 10 min. Injector temperature was 240°C. Mass spectrometer operated in scan mode over mass range from 33 to 300 amu (2 s/scan) at an ionization potential of 70 eV. Identification of volatile compounds was achieved by comparing mass spectra with the Wiley library (Wiley7, NIST 05). The volatile compounds were identified by matching the retention indices (RI) calculated according to the equation of Van Den Dool and Kratz (1963) and based on a series of alkanes. The data are expressed like relative peak area respect to internal standard. Blank experiments were conducted in two different modalities: blank of the fiber and blank of the empty vial. These types of control were carried out every 4 analyses. All analyses were performed in duplicate.

### Statistical analysis

All statistical analyses (Analysis of Variance (ANOVA); Principal Component Analysis) and graphs were performed using SYSTAT 13.0 for Windows (Systat Software Inc., Richmond, CA, USA).

## Results

### Dough acidification of fermented sourdough

All the flours (W, Q, K, and blend WQK) were microbiologically and chemically characterized (Table 1). Samples showed low counts of LAB and yeasts ranging between 1.2 (W) and 2.3 (Q) log CFU/g and 1.2 (W) and 1.9 (Q) CFU/g respectively. TTA values of quinoa samples were significantly different from the other ones. Quinoa samples were characterized by the highest TTA value (4.1 mL) compared to the other samples. pH values were very similar among the samples, only the sample W showed a significant difference compared with the other samples (Q, K, and WQK).

**Table 1:** Microbial and chemical characteristics of the flours used in the experiments.

**Flour samples**	**Microbial group (log CFU/g)**					**Chemical composition g/100g d.w**.					
	**TMC**	**LAB**	**Yeast**	**pH**	**TTA**	**Proteins**	**Fat**	**Ash**	**Fiber**	Carbohydrates^*^	**Moisture**
W	2.3 ± 0.4^a^	1.2 ± 0.1^a^	1.2 ± 0.1^a^	6.0 ± 0.2^a^	1.2 ± 0.1^a^	13.4 ± 0.20^a^	1.2 ± 0.02^a^	0.70 ± 0.01^a^	3.4 ± 0.21^a^	81.4	14.2 ± 0.05^a^
Q	2.6 ± 0.1^a^	2.3 ± 0.2^b^	1.9 ± 0.0^b^	6.5 ± 0.1^b^	4.1 ± 0.2^b^	14.6 ± 0.16^b^	6.2 ± 0.10^b^	3.17 ± 0.12^b^	12.0 ± 1.02^b^	64.0	8.4 ± 0.02^b^
K	1.7 ± 0.0^b^	1.7 ± 0.1^c^	1.2 ± 0.1^a^	6.4 ± 0.1^b^	1.2 ± 0.1^a^	14.9 ± 0.14^b^	2.1 ± 0.04^c^	1.80 ± 0.02^c^	9.0 ± 0.85^c^	71.2	10.9 ± 0.1^c^
WQK	2.6 ± 0.1^a^	2.1 ± 0.1^b^	1.5 ± 0.1^c^	6.4 ± 0.1^b^	1.9 ± 0.2^c^	14.5 ± 0.08^b^	2.8 ± 0.04^d^	1.88 ± 0.05^c^	8.7 ± 0.78^c^	72.1	11.1 ± 0.02^d^

*W, wheat flour; Q, quinoa flour, K, kamut flour; WQK, blend of wheat (25%), quinoa (25%) and kamut (50%) flours*.

*TMC, total mesophilic count; TTA, total titratable acidity*.

* *Calculated by difference*.

*Different superscript letters (a,b,c,d) between means within a column indicate statistically significant differences (p < 0.05) among different flours used*.

Regarding chemical composition, quinoa (Q) and kamut (K) samples showed fiber (12.0 and 9.0 g/100 g d.w., respectively) and proteins content (14.6 and 14.9 g/100 g d.w. respectively) significantly higher than wheat flour (W). Quinoa flour evidenced a greatest value of fat (6.2 g/100 g d.w.) and ash (3.17 g/100 g d.w.) compared to the other samples.

**Figure 1:**
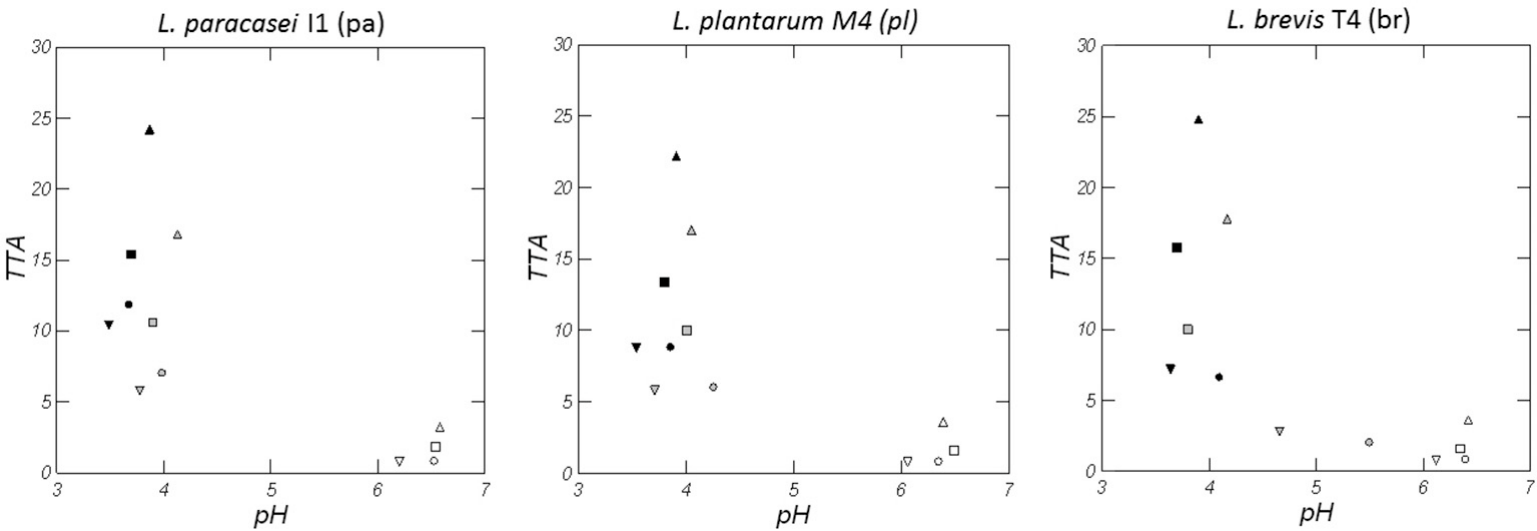
pH and TTA variation of doughs of Wheat, W (▿, ▾, ▾), Quinoa, Q (△, ▴, ▴), Kamut, K (○, ●, ●), and mixture of the different flours, WQK (□, ■, ■) fermented by *L. paracasei* I1 (pa), *L. plantarum* M4 (pl), and *L. brevis* T4 (br) at time zero (open symbols), after 24 h (gray symbols) and 48 h (full symbols). Results are shown as mean of two determinations. Standard deviation of pH values ranged from 0.01 to 0.35 while standard deviation of TTA values ranged from 0.14 to 0.71).

Figure 1 shows pH and TTA values of the doughs prepared with wheat (W), quinoa (Q), Kamut® (K), and a blend of flours (WQK) fermented for 48 h by *L. paracasei* I1 (pa), *L. plantarum* M4 (pl) and *L. brevis* T4 (br) strains. At time zero all the doughs obtained with wheat (W) had a pH value of about 6.1 and a TTA value of about 0.8 mL, whereas the doughs Q, K and WQK had a pH value of about 6.4 and a TTA value between 0.8 mL ± 0.07 (K samples) and 3.5 mL ± 0.21 (Q samples). During fermentation all the doughs showed a high acidification reaching after 48 h a pH value below 4.0, although considerable differences in TTA values were recorded among the samples. After 48 h, in fact, the TTA reached values comprised between 22.2 ± 0.51 mL (Qpl) and 25.0 ± 0.62 mL (Qbr) in the doughs prepared with quinoa flour and comprised between 13.4 ± 0.23 mL (WQkpl) and 15.8 ± 0.11 mL (WQKbr) in the doughs prepared with the blend of the flours (WQK samples). Instead, all the doughs prepared with wheat and Kamut® flours, after 48 h fermentation, showed a value of TTA below 12.0 mL.

### Lactic acid bacteria growth

**Figure 2:**
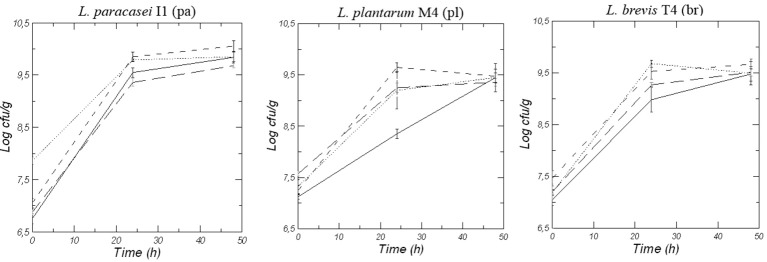
Viable counts of LAB in doughs of Wheat, W (-- --), Quinoa, Q (------), Kamut, K (-—) and mix of the different flours, WQK (···) fermented for 48 h by *L. paracasei* I1 (pa), *L. plantarum* M4 (pl), and *L. brevis* T4 (br). Results are shown as mean ± standard deviation.

Figure 2 shows the viable counts of lactic acid bacteria strains during 48 h of fermentation of doughs made with wheat (W), quinoa (Q), Kamut® (K) and blend of the three flours (WQK). After an initial inoculum of about 7–7.5 log CFU/g, an increase in microbial growth of each starter strain (*L. paracasei* I1, *L. plantarum* M4, and *L. brevis* T4) was recorded for all the conditions. After 24 h of fermentation all the strains well adapted to the different flours with counts higher than 9.0 log CFU/g even if the best growth was recorded in the doughs made with quinoa (Q and WQK samples) respect to doughs made with wheat (W samples) and Kamut® (K samples). Among all the used strains, *Lactobacillus paracasei* I1 was better adapted to quinoa matrix and persisted during quinoa sourdough fermentation achieved a value of 10.0 log CFU/g after 48 h fermentation.

### Volatile organic compounds formation

Volatile organic compounds (VOCs) were analyzed in the doughs during fermentation and using the doughs at time zero as the reference. More than 70 volatile components, belonging to different chemical classes, were identified through SPME-GC/MS. Peaks with area <1% of the total peak areas and with no significant differences (ANOVA, Tukey's HSD test) in the different conditions were discarded from further statistical and graphical analyses.

The main differences were evidenced after 48 h of fermentation. **Table 3** shows the forty-nine volatile components that mainly (*p* < 0.05) (ANOVA) characterized and differentiated doughs after 48 h of fermentation. The most characteristic ones belonged to eight classes such as, aldehydes, sulfur compounds, ketones, esters and acetates, alcohols, furans, pyrazines and acids.

Low aldehydes compounds was observed in all the samples during fermentation. In particular the doughs prepared with Kamut® flour (K and WQK samples) were characterized after 48 h of fermentation by pentanal, hexanal, trans (E)2-heptanal, nonanal, 2-octenal, 2,4-nonadienal and 2,4-decadienal, mainly when fermented by *L. plantarum* M4 and *L. paracasei* I1. Instead, at time zero doughs prepared with quinoa flour (Qc and WQKc) were characterized by high amount of benzaldehyde, 2-methylbutanal and 3-methylbutanal which during fermentation have decreased.

Sulfur compounds were not detectable in the samples at time zero but after 48 h fermentation the compound dimethylsulfide was recorded mainly in the sample prepared with quinoa flour (Qpa, Qbr, Qpl).

The doughs at time zero were characterized by low amount of ketones. Diacetyl and acetoin were not detectable at time zero but increased during fermentation in all the samples, mainly in those prepared with quinoa flour and fermented with *L. paracasei* I1 (Qpa) and *L. brevis* T4 (Qbr).

Furthermore, *L. paracasei* I1, compared to *L. brevis* T4 and *L. plantarum* M4 strains, produced the highest amount of 2,6-dimethyl-4-heptanone, 5-methyl-3-hexanone, 4-methyl-3-penten-2-one, volatile compounds. The highest amount were recorded in the wheat and Kamut® flours after 48 h of fermentation (Wpa, Kpa, WQKpa).

Considerable amount of 2-butanone and 3,5-octadien-2-one were detected only in the quinoa samples at time zero (Qc) and after 48 h fermentation (Qpa, Qbr, Qpl). During fermentation a considerable production of 3-octanone, 2-octanone, 1-octen-3-one and 3-octen-2-one were recorded in the samples containing quinoa flour (Q and WQK samples).

Among esters and acetates, the ethyl acetate was the sole compound found in appreciable amount during dough fermentation mainly in the doughs fermented by *L. brevis* T4 (Wbr, Kbr, WQKbr).

A considerable production of various alcohols was recorded during fermentation. As expected, the highest amount of ethanol was found in the doughs fermented by the hetero-fermentative lactic acid bacteria, *L. brevis* T4. Some alcohols characterized doughs prepared with Kamut® flour such as, 1-hexanol, 1-octen-3-ol, 1-heptanol, 1-octanol, 1-nonanol, others have characterized doughs prepared with quinoa flour such as, 2-furanmethanol, benzyl alcohol and 2-phenylethanol.

Among furans, 2-ethylfuran and 2-pentylfuran prevailed mainly in the doughs prepared with Kamut® after 48 h fermentation (K and WQK).

Pyrazines were found only in the doughs prepared with quinoa flour (Q and WQK) both at time zero and after fermentation. The more noticeable compounds found were methyl pyrazine, 2,6-dimethyl pyrazine, 2-ethyl-5-methyl pyrazine, 2,3,5-trimethyl pyrazine.

Sourdoughs at time zero were characterized by absence or irrelevant amount of organic acids. After 48 h fermentation, instead, all the doughs achieved a strong acidification, mainly those fermented by *L. brevis* T4. As shown in Figure 1 the highest TTA values were recorded for samples produced with quinoa flour. Among volatile acids detected by SPME, the main compounds found were acetic, hexanoic, 2-methylbutanoic and pentanoic acid. In detail, acetic and hexanoic acids were found in all the samples while 2-methylbutanoic and pentanoic acids were found mainly in the samples prepared with quinoa flour (Q, WQK).

**Figure 3:**
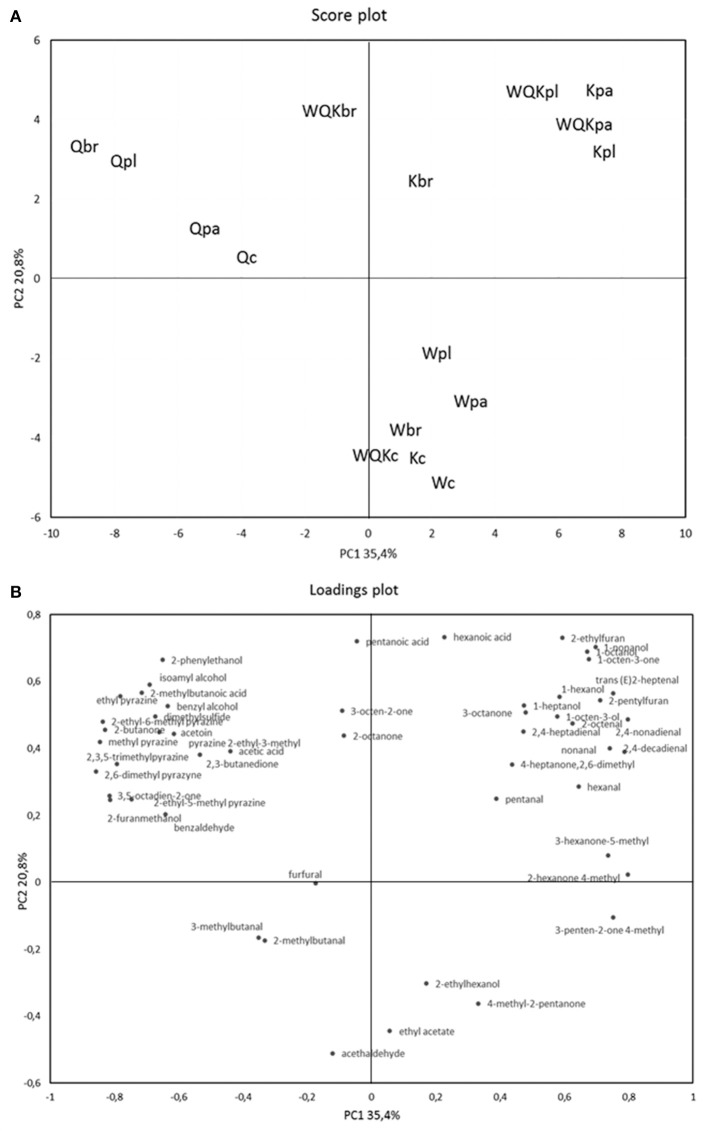
Score plot **(A)** and loading plot **(B)** of first and second principal components after principal-component analysis based on volatile components that mainly (*P* < 0.05) differentiated the four sourdoughs: Q (quinoa), K (kamut), W (wheat), and WQK (mix) fermented by *L. paracasei* (pa), *L. plantarum* (pl), *L. brevis* (br) after 48 h. Volatile organic compounds used in PCA are listed in Table 2.

In order to better understand the differences among the dough samples, a PCA of the 49 volatile compounds recorded after 48 h fermentation of the different doughs was calculated as shown in Figures 3A,B. The two PCs explained ca. 56.2% of the total variance of the data. Doughs prepared with different flours, as determined by the two PCs (factors), were located in different zones of the plane. Regarding the scores plot, there is a clear separation between quinoa doughs, Q (negative component of the PC1) and wheat, Kamut® and the blend of the different flours (positive component of the PC1).

**Table 2:** Concentrations of volatile components identified in the doughs a time zero (control samples) and after 48 h fermentation.

**RI**	**Compounds**	**Control samples**				**Fermentation by** ***L. paracasei*** **I1**				**Fermentation by** ***L. plantarum*** **M4**				**Fermentation by** ***L. brevis*** **T4**				**Odor^#^**
		**Wc**	**Qc**	**Kc**	**WQKc**	**Wpa**	**Qpa**	**Kpa**	**WQKpa**	**Wpl**	**Qpl**	**Kpl**	**WQKpl**	**Wbr**	**Qbr**	**Kbr**	**WQKbr**	
**ALDEHYDES**																		
719	Acethaldehyde	0.8 ± 0.1^a^	1.3 ± 0.1^bc^	1.1 ± 0.1^b^	1.4 ± 0.0^c^	nd	0.3 ± 0.0^a*^	0.2 ± 0.0^b*^	0.2 ± 0.0^b*^	0.1 ± 0.0^a*^	0.2 ± 0.0^b^°	0.2 ± 0.0^b*^	0.5 ± 0.2^c^°	nd	nd	0.2 ± 0.0^a*^	nd	Fruity
970	2-methylbutanal	nd	14.1 ± 0.1^a^	0.31 ± 0.0^b^	3.2 ± 0.1^c^	nd	0.5 ± 0.0^a*^	nd	0.4 ± 0.0^b*^	nd	0.1 ± 0.0^a^°	nd	0.2 ± 0.0^a^°	nd	0.1 ± 0.0^a^°	nd	nd	Fruity
976	3-methylbutanal	nd	15.8 ± 0.3^a^	0.22 ± 0.0^b^	3.5 ± 0.1^c^	nd	0.9 ± 0.3^a*^	0.1 ± 0.0^b*^	0.3 ± 0.0^c*^	nd	0.3 ± 0.0^a^°	nd	0.1 ± 0.0^b^°	nd	0.2 ± 0.0^a§^	0.2 ± 0.0^a*^	nd	Almond
960	Pentanal	0.3 ± 0.03^a^	0.5 ± 0.0^b^	0.66 ± 0.1^c^	2.0 ± 0.1^d^	nd	0.4 ± 0.0^a*^	4.6 ± 0.1^b*^	5.4 ± 0.5^c*^	1.7 ± 0.0^a*^	0.4 ± 0.0^b*^	3.0 ± 0.2^c^°	2.6 ± 0.1^d^°	nd	nd	1.7 ± 0.1^a§^	nd	Almond, malt
1108	Hexanal	8.8 ± 0.2^a^	1.7 ± 2.2^b^	12.2 ± 0.4^c^	19.2 ± 1.2^d^	15.0 ± 0.1^a*^	1.3 ± 0.0^b*^	57.7 ± 1.1^c*^	50.4 ± 1.2^d*^	48.9 ± 1.9^a^°	3.5 ± 0.1^b^°	65.7 ± 3.6^c^°	49.2 ± 1.3^a*^	8.0 ± 0.3^a§^	nd	13.5 ± 1.0^b§^	4.3 ± 0.2^c^°	Green
1332	trans (E)2-heptenal	nd	nd	nd	nd	3.6 ± 0.3^a*^	1.3 ± 0.0^b*^	30.7 ± 1.4^c*^	25.1 ± 1.4^d*^	7.2 ± 0.1^a^°	2.9 ± 0.0^b^°	33.2 ± 1.4^c*^	35.6 ± 0.7^d^°	3.0 ± 0.1^a§^	nd	6.6 ± 0.4^b^°	0.8 ± 0.0^c§^	Green
1398	Nonanal	nd	2.5 ± 0.1^a^	0.7 ± 0.0^b^	0.5 ± 0.0^c^	nd	nd	1.7 ± 0.1^a*^	1.4 ± 0.0^b*^	0.6 ± 0.0^a*^	nd	2.4 ± 0.0^b^°	1.0 ± 0.0^c^°	nd	nd	nd	nd	Rose
1430	2-octenal	nd	nd	0.1 ± 0.0^a^	nd	4.2 ± 0.4^a*^	0.3 ± 0.0^b*^	41.5 ± 2.4^c*^	34.3 ± 1.2^d*^	11.8 ± 0.8^a^°	nd	1.7 ± 0.1^b^°	43.4 ± 1.6^c^°	3.3 ± 0.3^a§^	nd	7.1 ± 0.2^b§^	2.7 ± 0.1^c§^	Fatty, green
1520	Benzaldehyde	1.0 ± 0.1^a^	18.4 ± 4.2^b^	1.6 ± 0.1^c^	6.2 ± 0.3^d^	1.0 ± 0.0^a*^	15.4 ± 0.4^b*^	5.3 ± 0.1^c*^	5.4 ± 0.0^c*^	0.6 ± 0.0^a*^	4.4 ± 0.1^b^°	1.9 ± 0.3^c^°	1.3 ± 0.2^d^°	3.3 ± 0.2^a§^	12.5 ± 0.1^b§^	2.6 ± 0.2^c§^	3.5 ± 0.0^a§^	Almond, sweet
1713	2.4-nonadienal	nd	nd	nd	nd	0.8 ± 0.0^a*^	nd	2.6 ± 0.1^b*^	1.9 ± 0.1^c*^	1.4 ± 0.1^a^°	nd	3.1 ± 0.2^b^°	2.6 ± 0.2^c^°	0.3 ± 0.0^a§^	nd	0.5 ± 0.0^b§^	nd	Fatty, green, cucumber
1777	2.4-decadienal	nd	nd	nd	nd	0.7 ± 0.1^a*^	nd	2.5 ± 0.2^b*^	1.3 ± 0.0^c*^	0.5 ± 0.0^a^°	nd	2.0 ± 0.3^b*^°	1.4 ± 0.2^c*^	0.6 ± 0.0^a*^	nd	1.8 ± 0.1^b^°	0.8 ± 0.0^c^°	Citrus, green
**SULFUR COMPOUNDS**																		
767	Dimethylsulfide	nd	nd	nd	nd	0.2 ± 0.0^a*^	1.2 ± 0.1^b*^	0.1 ± 0.0^c*^	0.2 ± 0.0^a*^	0.1 ± 0.0^a^°	1.0 ± 0.0^b^°	nd	0.2 ± 0.0^c*^	0.2 ± 0.0^a*^	0.9 ± 0.0^b§^	0.1 ± 0.0^c*^	0.3 ± 0.0^d^°	Cabbage, Vegetable
**KETONES**																		
963	Diacetyl	nd	nd	nd	nd	27.9 ± 0.2^a*^	87.4 ± 0.9^b*^	23.6 ± 1.5^c*^	6.7 ± 0.3^d*^	2.2 ± 0.1^a^°	9.4 ± 0.1^b^°	1.0 ± 0.0^c^°	0.8 ± 0.0^d^°	0.3 ± 0.0^a§^	80.3 ± 8.9^b*^	7.4 ± 0.6^c§^	12.6 ± 0.2^d§^	Butter, fatty
999	4-methyl-2-pentanone	10.3 ± 0.9^abc^	9.4 ± 0.7^ab^	11.2 ± 0.9^ac^	20.1 ± 1.5^d^	14.8 ± 0.6^a*^	13.7 ± 0.4^b*^	13.3 ± 1.3^ab*^	14.8 ± 0.5^a*^	7.1 ± 0.2^a^°	6.5 ± 0.5^a^°	12.7 ± 0.0^b^°	11.5 ± 0.7^c^°	23.2 ± 1.4^a§^	7.3 ± 0.1^b§^	9.1 ± 0.1^c§^	7.5 ± 0.4^b§^	Green
1006	5-methyl-3-hexanone	6.3 ± 0.4^a^	3.5 ± 0.1^b^	3.1 ± 0.2^b^	4.9 ± 0.1^c^	7.7 ± 0.2^a*^	5.9 ± 0.4^b*^	7.3 ± 0.0^c*^	7.5 ± 0.8^ac*^	4.7 ± 0.2^a^°	3.0 ± 0.2^b^°	6.5 ± 0.3^c^°	5.6 ± 1.1^d^°	5.7 ± 0.2^a§^	2.6 ± 0.2^b^°	4.8 ± 0.0^c§^	5.4 ± 0.2^a^°	Fruity
908	2-butanone	nd	2.4 ± 0.1^a^	nd	0.3 ± 0.0^b^	nd	4.1 ± 0.2^a*^	nd	nd	nd	3.1 ± 0.2^a^°	nd	1.1 ± 0.1^b*^	nd	4.2 ± 0.1^a*^	nd	0.9 ± 0.0^b^°	Fruity, green
1124	4-methyl-2-hexanone	16.9 ± 0.6^a^	9.2 ± 0.9^b^	17.2 ± 1.1^a^	14.2 ± 1.2^d^	26.7 ± 1.8^a*^	16.9 ± 0.4^b*^	25.3 ± 1.1^a*^	24.2 ± 1.3^a*^	14.3 ± 1.2^a^°	9.3 ± 0.9^b^°	20.7 ± 0.6^c^°	17.8 ± 0.7^d^°	15.2 ± 1.1^a^°	7.8 ± 0.2^b§^	15.3 ± 1.2^a§^	12.0 ± 0.4^c§^	Fruity
1140	4-methyl-3-penten-2-one	39.9 ± 3.9^a^	21.9 ± 0.2^b^	38.8 ± 0.3^a^	27.4 ± 1.2^d^	50.7 ± 0.7^a*^	26.7 ± 1.6^b*^	43.7 ± 1.7^c*^	52.3 ± 1.2^a*^	27.0 ± 0.3^a^°	13.6 ± 0.9^b^°	39.3 ± 1.2^c^°	31.0 ± 0.3^d^°	21.6 ± 0.7^a§^	11.5 ± 1.0^b§^	22.6 ± 0.8^a§^	22.3 ± 1.2^a§^	vegetable
1210	2,6-dimethyl-4-heptanone	11.4 ± 0.6^a^	7.0 ± 0.1^b^	2.3 ± 0.2^c^	6.4 ± 0.0^d^	77.5 ± 13.8^a*^	1.4 ± 0.1^b*^	162.7 ± 7.8^c*^	210.2 ± 2.7^d*^	2.0 ± 0.0^a^°	70.1 ± 1.7^b^°	4.6 ± 0.1^c^°	2.5 ± 0.2^d^°	2.4 ± 0.1^a§^	2.1 ± 0.0^b§^	1.5 ± 0.3^c§^	7.6 ± 0.4^d§^	Fruity
1262	3-octanone	nd	nd	nd	nd	nd	0.6 ± 0.0^a*^	nd	6.1 ± 0.3^b*^	nd	1.0 ± 0.0^a§^	4.6 ± 0.0^b*^	6.3 ± 0.3^c*^	nd	0.8 ± 0.0^a*^	1.1 ± 0.1^b^°	0.3 ± 0.0^c^°	Herbal, mushr.
1283	2-octanone	nd	0.7 ± 0.0^a^	nd	0.4 ± 0.0^a^	nd	0.5 ± 0.0^a*^	nd	0.9 ± 0.0^b*^	nd	0.8 ± 0.1^a*^	1.1 ± 0.1^b*^	0.8 ± 0.0^a^°	nd	0.7 ± 0.0^a§^	0.1 ± 0.0^b^°	4.1 ± 0.1^c§^	Woody, herbal
1295	Acetoin	nd	nd	nd	nd	16.7 ± 0.8^a*^	71.8 ± 2.0^b*^	9.9 ± 0.1^c*^	4.4 ± 0.3^d*^	8.0 ± 0.1^a^°	51.7 ± 1.6^b^°	2.9 ± 0.0^c^°	5.9 ± 0.2^d^°	4.2 ± 0.1^a§^	57.0 ± 1.7^b§^	6.6 ± 0.1^c§^	2.4 ± 0.1^d§^	Butter, fatty
1311	1-octen-3-one	nd	nd	nd	nd	nd	0.4 ± 0.0^a*^	2.3 ± 0.2^b*^	2.3 ± 0.2^b*^	1.0 ± 0.1^a*^	1.0 ± 0.1^a^°	2.4 ± 0.0^b*^	2.8 ± 0.1^c^°	0.7 ± 0.0^a§^	nd	0.8 ± 0.0^b^°	1.3 ± 0.0^c§^	Herbal, mushr.
1416	3-octen-2-one	nd	1.8 ± 0.1^a^	nd	0.4 ± 0.0^b^	1.2 ± 0.1^a*^	1.4 ± 0.1^a*^	4.4 ± 0.3^b*^	0.2 ± 0.0^c*^	nd	0.8 ± 0.3^a^°	nd	0.6 ± 0.0^a^°	nd	1.9 ± 0.2^a§^	nd	3.0 ± 0.0^b§^	Herbal, mushr.
1581	3,5-octadien-2-one	nd	0.8 ± 0.0^a^	nd	nd	nd	0.4 ± 0.0^a*^	nd	nd	nd	0.5 ± 0.0^a^°	nd	nd	nd	0.5 ± 0.0^a^°	nd	nd	Fruity, fatty, mush.
**ESTERS AND ACETATES**																		
889	Ethyl acetate	nd	nd	nd	nd	nd	nd	nd	0.2 ± 0.0^a*^	nd	nd	0.3 ± 0.0^a*^	0.4 ± 0.0^b^°	6.2 ± 0.1^a*^	nd	5.5 ± 0.4^b^°	0.7 ± 0.0^c§^	Fruity, sweet
**ALCHOLS**																		
	Ethanol	nd	nd	nd	nd	1.7 ± 0.3^a*^	0.4 ± 0.0^b*^	1.3 ± 0.1^a*^	0.9 ± 0.0^c*^	0.1 ± 0.0^a^°	1.2 ± 0.1^b^°	1.6 ± 0.1^c^°	0.9 ± 0.3^b*^	101.71 ± 0.4^a§^	131.26 ± 0.4^b§^	112.67 ± 0.3^c§^	90.25 ± 0.1^d^°	Alcoholic
1184	Isoamyl alcohol	0.4 ± 0.03^a^	1.1 ± 0.1^b^	0.6 ± 0.0^c^	0.7 ± 0.0^d^	nd	9.8 ± 0.3^a*^	2.0 ± 0.1^b*^	4.3 ± 0.0^c*^	0.5 ± 0.0^a*^	18.2 ± 1.5^b^°	0.8 ± 0.0^c^°	3.1 ± 0.1^d^°	0.7 ± 0.0^a^°	15.2 ± 0.1^b§^	1.6 ± 0.1^c§^	3.6 ± 0.3^d§^	Alcoholic, fruity
1361	1-hexanol	6.2 ± 0.8^a^	8.5 ± 0.6^b^	17.5 ± 0.5^c^	12.7 ± 0.3^d^	14.1 ± 0.2^a*^	25.7 ± 1.5^b*^	74.1 ± 3.0^c*^	55.0 ± 1.7^d*^	54.7 ± 0.7^a^°	39.7 ± 0.7^b^°	191.5 ± 9.5°^c^	158.1 ± 2.9^d^°	12.9 ± 0.5^a§^	21.6 ± 1.3^b§^	31.3 ± 1.2^c§^	39.8 ± 0.5^d§^	Sweet, green
1450	1-octen-3-ol	0.98 ± 0.08^a^	2.3 ± 0.1^b^	0.9 ± 0.0^a^	1.0 ± 0.1^a^	6.1 ± 0.2^a*^	3.5 ± 0.1^b*^	27.7 ± 0.7^c*^	17.9 ± 0.4^d*^	3.8 ± 0.2^a^°	4.9 ± 0.1^b^°	69.5 ± 1.0^c^°	16.9 ± 0.4^d*^	6.1 ± 0.0^a*^	5.2 ± 0.2^b^°	7.9 ± 0.2^c§^	21.7 ± 0.6^d^°	Mushroom, earthy
1453	1-heptanol	nd	0.8 ± 0.0^a^	nd	nd	1.5 ± 0.2^a*^	2.0 ± 0.2^b*^	6.9 ± 0.4^c*^	nd	2.1 ± 0.3^a^°	2.4 ± 0.1^a^°	18.1 ± 1.6^b^°	7.7 ± 0.2^c*^	0.8 ± 0.0^a§^	1.5 ± 0.1^b§^	1.0 ± 0.0^c§^	5.9 ± 0.2^d^°	Sweet, woody
1565	1-octanol	nd	0.5 ± 0.1^a^	0.4 ± 0.0^a^	0.4 ± 0.0^a^	2.6 ± 0.2^a*^	1.3 ± 0.0^b*^	7.1 ± 0.2^c*^	7.5 ± 0.7^c*^	2.4 ± 0.1^a*^	1.7 ± 0.1^b^°	7.9 ± 0.1^c^°	7.1 ± 0.1^c*^	1.3 ± 0.1^a^°	1.3 ± 0.2^ab*^	1.7 ± 0.3^b§^	6.9 ± 0.2^c*^	Green, orange
1668	1-nonanol	nd	nd	nd	nd	1.2 ± 0.0^a*^	0.7 ± 0.0^b*^	3.2 ± 0.1^c*^	2.9 ± 0.6^c*^	1.2 ± 0.0^a*^	0.7 ± 0.1^b*^	3.2 ± 0.1^c*^	3.3 ± 0.3^c*^	0.4 ± 0.0^a^°	0.4 ± 0.0^a^°	0.6 ± 0.0^b^°	2.2 ± 0.4*c*^b*^	Floral
1673	2-furanmethanol	nd	1.2 ± 0.1^a^	nd	nd	nd	nd	nd	nd	nd	0.8 ± 0.0^a*^	nd	nd	nd	1.0 ± 0.1^a^°	nd	nd	Sweet, Caramellic
1889	Benzyl alcohol	nd	0.5 ± 0.0^a^	nd	nd	nd	0.9 ± 0.1^a*^	nd	0.3 ± 0.0^b*^		2.9 ± 0.0^a^°	nd	0.7 ± 0.0^b^°	nd	1.4 ± 0.1^a§^	nd	1.3 ± 0.1^a§^	Floral, rose
1925	2-phenylethanol	nd	0.3 ± 0.0^a^	nd	nd	nd	0.6 ± 0.0^a*^	0.2 ± 0.1^b*^	0.3 ± 0.1^b*^	0.2 ± 0.0^a*^	1.5 ± 0.1^b^°	0.2 ± 0.0^a*^	0.4 ± 0.0^c*^	0.2 ± 0.0^a*^	1.1 ± 0.0^b§^	nd	0.4 ± 0.0^c*^	Floral, sweet
**FURANS**																		
950	2-ethylfuran	nd	1.3 ± 0.1^a^	0.5 ± 0.0^b^	0.3 ± 0.0^c^	nd	1.2 ± 0.1^a*^	7.2 ± 0.1^b*^	6.4 ± 0.4^c*^	1.5 ± 0.2^a*^	1.1 ± 0.2^a*^	5.2 ± 0.1^b^°	5.3 ± 0.4^b^°	nd	1.2 ± 0.0^a*^	0.7 ± 0.0^b§^	4.5 ± 1.1^c^°	Burnt, malty
1241	2-pentylfuran	2.04 ± 0.08^a^	9.8 ± 0.2^b^	2.8 ± 0.0^a^	0.8 ± 0.1^c^	37.9 ± 0.3^a*^	10.2 ± 0.6^b*^	94.2 ± 5.6^c*^	53.5 ± 0.9^d*^	29.8 ± 0.4^a^°	13.2 ± 0.4^b^°	59.9 ± 0.3^c^°	44.4 ± 1.1^d^°	9.3 ± 1.1^a§^	11.2 ± 1.0^a*^	46.6 ± 0.5^b§^	36.7 ± 0.6^c§^	Green, vegetable
**PYRAZINES**																		
1274	Methyl pyrazine	nd	5.5 ± 0.0^a^	nd	0.6 ± 0.0^b^	nd	3.8 ± 0.2^a*^	nd	1.2 ± 0.1^b*^	nd	5.2 ± 0.1^a^°	nd	0.9 ± 0.1^b^°	nd	5.9 ± 0.1^a§^	nd	1.1 ± 0.1^b^°*	Nutty, roasted
1336	2.6-dimethyl pyrazine	nd	3.5 ± 0.1^a^	nd	nd	nd	2.0 ± 0.1^a*^	nd	nd	nd	3.3 ± 0.3^a^°	nd	nd	nd	3.1 ± 0.1^a^°	nd	0.1 ± 0.0^b*^	Nutty, bready
1343	Ethyl pyrazine	nd	1.0 ± 0.0^a^	nd	nd	nd	2.0 ± 0.1^a*^	nd	1.0 ± 0.0^b*^	nd	3.1 ± 0.3^a^°	nd	nd	nd	2.7 ± 0.1^a^°	nd	1.4 ± 0.1^b^°	Nutty, roasted
1393	2-ethyl-6-methylpyrazine	nd	1.5 ± 01^a^	nd	0.3 ± 0.0^b^	nd	1.1 ± 0.3^a*^	nd	0.3 ± 0.1^b*^	nd	2.5 ± 0.1^a^°	nd	0.3 ± 0.0^b*^	nd	1.7 ± 0.1^a§^	nd	0.9 ± 0.0^b^°	Roasted, potato
1395	2-ethyl-5-methylpyrazine	nd	2.0 ± 01^a^	nd	0.5 ± 0.0^b^	nd	1.1 ± 0.1^a*^	nd	0.2 ± 0.0^b*^	nd	0.8 ± 0.1^a^°	nd	nd	nd	2.3 ± 0.1^a§^	nd	0.2 ± 0.0^b*^	Roasted, coffee
1412	2,3,5-trimethylpyrazine	nd	2.0 ± 02^a^	nd	0.6 ± 0.0^b^	nd	1.4 ± 0.1^a*^	nd	0.4 ± 0.0^b*^	nd	1.0 ± 0.2^a^°	nd	0.5 ± 0.2^b^°*	nd	1.6 ± 0.1^a*^	nd	0.6 ± 0.0^b^°	Nutty, musty
1413	2-ethyl-3-methylpyrazine	nd	0.9 ± 0.0^a^	nd	0.1 ± 0.0^b^	nd	0.8 ± 0.0^a*^	nd	nd	nd	1.8 ± 0.1^a^°	nd	nd	nd	2.2 ± 0.1^a§^	nd	3.4 ± 0.1^b*^	Nutty, peanut
**ACIDS**																		
1459	Acetic acid	nd	nd	nd	nd	40.3 ± 4.2^a*^	78.7 ± 0.7^b*^	44.7 ± 1.5^a*^	25.3 ± 1.2^c*^	67.2 ± 2.8^a^°	47.0 ± 1.6^b^°	45.3 ± 1.3*^b^	51.1 ± 1.5^c^°	115.3 ± 4.1^a§^	108.9 ± 1.4^b§^	100.6 ± 2.4^c^°	100.4 ± 0.8^c§^	Acidic, fruity
1694	2-methylbutanoic acid	nd	nd	nd	nd	nd	3.7 ± 0.2^a*^	nd	1.2 ± 0.1^b*^	nd	4.3 ± 0.1^a^°	nd	0.8 ± 0.0^b^°	nd	6.1 ± 0.2^a§^	nd	1.5 ± 0.0^b§^	Butter, cheesy
1761	Pentanoic acid	nd	nd	nd	nd	nd	2.1 ± 0.1^a*^	4.0 ± 0.3^b*^	1.0 ± 0.0^c*^	1.6 ± 0.2^a*^	2.5 ± 0.0^b^°	nd	3.4 ± 0.1^c^°	1.0 ± 0.4^a*^	2.5 ± 0.1^b^°	nd	4.9 ± 0.3^c§^	Acidic, cheesy
1868	Hexanoic acid	nd	1.9 ± 0.2^a^	nd	0.2 ± 0.0^b^	42.0 ± 3.1^a*^	34.5 ± 0.9^b*^	94.4 ± 5.6^c*^	60.4 ± 1.2^d*^	13.6 ± 0.4^a^°	41.4 ± 1.7^b^°	32.3 ± 1.3^c^°	22.5 ± 1.7^d^°	22.2 ± 1.1^a§^	43.4 ± 1.5^b^°	22.6 ± 1.3^a§^	70.8 ± 0.4^c§^	Fatty, cheesy

# *based on online databases (www.flavornet.org, and www.thegoodscentscompany.com)*.

*nd, Not detected*.

*RAP, Relative Peak Area (Area Peak Compound/Area Peak Internal Standard) × 100 (RAP ± SD)*.

*RI, Retention Index, identification by comparison with RI database*.

*Lowercase letters (a, b, c, d) indicate significant differences (p < 0.05) in relative volatile compounds among different flours used*.

Symbols (^*^, °, ^§^) indicate significant differences (p < 0.05) in relative volatile compounds among different strains used.

In fact, according to factor 1 (35.4%), doughs made with quinoa flour were distributed oppositely to the doughs prepared with the other flours. Quinoa doughs fermented by lactic acid bacteria (Qpa, Qpl, Qbr) were located more distant respect to quinoa dough control (Qc). According to factor 2 (20.8%), sourdoughs at time zero (Wc, Kc, WQKc,) and wheat sourdoughs fermented by starter (Wpa, Wpl, Wbr) were separated from fermented sourdoughs made by Kamut® and quinoa and fermented by microbial starter (pa, pl, br).

Production of VOCs was poor in the control samples (Qc, Wc, Kc, WQKc) and in the doughs made by wheat (Wpl, Wpa, Wbr) even if fermented by lactic acid bacteria (lower right section of the graph), while the doughs made by quinoa (upper left section of the graph), Kamut® and blend of flours (upper right section of the graph) were clearly separated each other indicating a different and higher content of VOCs. Obviously, the doughs made with the mixed flours, whereas the 50% was constituted by Kamut®, were separated in the same section of the doughs made with the sole Kamut®.

Regarding the loadings plot, quinoa fermented doughs were distinguished by the highest contents in 1-heptanol, 3-octen-2-one, benzaldehyde, dimethylsulfide, diacetyl, 2-butanone, 2,6-dimethyl-4-heptanone, acetoin, 2-methylbutanoic acid, acetic acid, 2-phenylethanol. Doughs K and WQK were characterized by the highest content in hexanal, trans (E)2-heptenal, nonanal, 2,4-decadienal, isoamyl alcohol, 4-methyl-2-hexanone, 5-methyl-3-hexanone, 1-octen-3-one, 1-heptanol. Finally, wheat doughs were characterized by the highest contents of 4-methyl-2-pentanone, ethylacetate, 5-methyl-3-hexanone, 4-methyl-2-hexanone, 4-methyl-3-penten-2-one, 2,6-dimethyl-4-heptanone.

## Discussion

Flours were characterized by good microbiological and physico-chemical characteristics. Microbial contaminants were present at low levels and pH and TTA values were typical of the different flours. Quinoa and kamut flours were characterized by high protein and fiber content. Quinoa flour showed also the greatest amount of fat and ash content. These values were comparable to those reported in literature (Jancurová et al., 2009). The resulted blend WQK was characterized by a higher fiber content compared to W and K samples, useful to obtain a bakery product which, in according to EC Regulation (Regulation EC No. 1924/2006) on nutrition and health claims on food products, could be defined as “high fiber content” because it could contains at least 6 g of fiber per 100 g of product. This blend WQK had also an ameliorate nutritional value, respect to wheat flour, in terms of proteins and ash content.

During fermentation all the doughs showed high acidification although the greatest differences in TTA values were recorded for samples produced with quinoa flour. Also Vogelmann et al. (2009) evidenced that the highest TTA values were obtained for sourdoughs produced with the pseudo-cereals amaranth and quinoa, whereas the wheat, rice, buckwheat or cassava sourdoughs showed lower TTA values. These differences can be explained by the buffering capacity of the flours. In addition, the extraction rate of the flour is one the most important factors influencing the TTA of sourdough. As also evidenced by Hansen (2006) the final TTA in sourdoughs made from whole meal flour (ash content about 1.5%) is almost double compared to sourdoughs made from white flour (ash content about 0.55%). In our research TTA values were higher in the doughs made with quinoa (Q) or containing quinoa flour (WQK) respect to the doughs prepared with wheat or Kamut® flour alone. Quinoa flour used in this research was characterized, in fact, by an ash value of 3.17 g/100 g d.w. whereas Kamut® and wheat flours were characterized by an ash value of 0.7 g/100 g d.w. and 1.88 g/100 g d.w., respectively (Table 1). The acidification process of the doughs was almost strictly dependent on the kind of flours used and not strictly related to the microbial strain used. In fact, pH and TTA showed small variations with the use of *L. paracasei, L. plantarum* and *L. brevis* species although the last one reached an intensive acidification only after an initial adaptation to substrate. *L. brevis* T4, in fact, showed a slowest dough acidification both in wheat and Kamut® reaching after 24 h pH values of 4.8 ± 0.2 (Wbr) and 5.5 ± 0.5 (Kbr), respectively.

The acidification process, affected by the application of lactic acid bacteria starter or sourdough, is mainly used to improve quality, taste and flavor of wheat breads (Brümmer and Lorenz, 1991; Katina et al., 2006a; Arendt et al., 2007) and to delay staling (Katina et al., 2006b; Plessas et al., 2007). Depending on the level of lactic acidification, sourdough fermentation influences bread extensibility, softness, volume and activity of endogenous enzymes (Nionelli and Rizzello, 2016). However, a higher acidity could negatively affect bread quality since it can cause off-flavor and reduce loaf volume as well as diminish crumb softness suitable for optimal wheat bread preparation (Kaditzky et al., 2008). So, to obtain good qualitative characteristics of baked foods, the choice of the right culture starter is indispensable for an adequate degree of dough acidification.

In this case *L. paracasei* and *L. plantarum* strains could be recommended because their use drops the pH safely below 4.6 in the shortest time. The quick acidification will favor the inhibition of undesirable microorganisms in the dough. Furthermore, their use allowed to obtain in minor time matured doughs characterized by a pH of 3.8–4.2 and a total titratable acidity comprised between 6.0 and 16.0 mL.

Flours resulted a good matrix for the growth of all the strains. In fact, after an initial inoculum of about 7–7.5 log CFU/g, an increase in microbial growth of each starter (*L. paracasei* I1, *L. plantarum* M4 and *L. brevis* T4) was recorded for all the conditions. However, quinoa was the best growing condition for all the strains. The ability of these LAB species to grow well in quinoa doughs was also evidenced in a study of Ruiz Rodriguez et al. (2016) that showed that the species *L. plantarum* and *L. brevis* has been found to dominate laboratory quinoa sourdoughs from day 3 up to the end of fermentation. The prevalence of *L. plantarum* during daily propagation of sourdoughs was attributed to its versatile metabolism, the ability to adapt to different environmental conditions and its large antimicrobial spectrum (Minervini et al., 2010). On the other hand, *L. brevis* was found to predominate in wheat and maize/rye sourdough ecosystems (Minervini et al., 2012; Rocha and Malcata, 2012). Moreover, our study evidenced that also the species *L. paracasei* was easily adapted to quinoa matrix and persisted during quinoa sourdough fermentation. In fact, *L. paracasei* I1 achieved a value of 10.0 log CFU/g after 48 h fermentation resulting a good putative microorganisms starter for quinoa sourdough. In addition this strain was previously characterized for its good ability to cope with acid stress (Reale et al., 2015). The ability of lactic acid bacteria to growth better in quinoa flour is probably due to the qualitative characteristics of quinoa that is also noteworthy for its high protein content with a balanced composition of essential amino acids instead of wheat flour (Comai et al., 2007). Amino acid content of quinoa and wheat flour was compared (see Table 3). The amino acid score (AAS), useful to predict protein quality in terms of capacity of the food to provide the appropriate pattern of dietary indispensable amino acids, was higher in quinoa flour than wheat flour (AAS = 92 and 54 respectively for quinoa flour and wheat flour), essentially due to the significative lower content of the amino acid lysine in wheat flour than quinoa flour, highlighting the highest biological value of quinoa proteins.

**Table 3:** Amino acids content and Amino Acid Score (AAS) of quinoa and wheat flours.

**Amino acid (g/100 g protein)**	**Recommended amino acid scoring patterns for older children adolescents and adults (FAO, 2013)**	**Quinoa flour**	**Wheat flour**
Histidine	1.6	4.09 ± 0.20^a^	2.48 ± 0.15^b^
Isoleucine	3.0	3.02 ± 0.24^a^	3.37 ± 0.41^a^
Leucine	6.1	6.88 ± 0.01^a^	7.63 ± 0.18^b^
Lysine	4.8	6.30 ± 0.03^a^	2.58 ± 0.02^b^
Sulfur amino acids	2.3	3.66 ± 0.23^a^	3.95 ± 0.41^a^
Aromatic amino acids	4.1	8.18 ± 0.55^a^	8.86 ± 0.22^a^
Threonine	2.5	4.41 ± 0.06^a^	3.09 ± 0.14^b^
Valine	4.0	3.67 ± 0.23^a^	4.07 ± 0.22^a^
Tryptophan	0.6	1.18 ± 0.15^a^	1.06 ± 0.09^a^
AAS		92	54

*Different superscript letters (a, b) between means within a row indicate statistically significant differences (p < 0.05)*.

Furthermore, as evidenced by Dallagnol et al. (2011) lactic fermentation indirectly stimulated flour protein hydrolysis by endogenous proteases of the flours and quinoa protein hydrolysis is usually faster respect to wheat protein hydrolysis determining a higher amounts of peptides and free amino acids in quinoa dough compared to wheat dough. Definitely, the better microbial growth was recorded in quinoa (Q samples) and in the blend of flours (WQK samples) because most probably the high nutrition value of quinoa favors lactic acid bacteria, that are microorganisms fastidious from the nutritional point of view that require more nutrients (vitamins, amino acids, etc) to grow. Then, the supplementation of wheat flour with quinoa flour could enhance not only the nutritional quality of baked goods but also the growth and the fermentation activities of lactic acid bacteria. In conclusion, quinoa flour, owing to its high nutritional value, results in a optimal substrate of growth for lactic acid bacteria.

Moreover, many differences were found in the volatile composition of the fermented doughs.

The main differences were evidenced after 48 h of fermentation for forty-nine volatile components comprised of aldehydes, sulfur compounds, ketones, esters and acetates, alcohols, furans, pyrazines and acids. During fermentation a low production of aldehydes compounds was found. The only samples produced with kamut flour (K and WQK) and fermented with *L. plantarum* M4 and *L. paracasei* I1 showed high production of pentanal, hexanal, trans (E)2-heptanal, nonanal, 2-octenal, 2,4-nonadienal and 2,4-decadienal.

Among sulfur compounds, dimethylsulfide was recorded mainly in the sample prepared with quinoa flour (Qpa, Qbr, Qpl). The presence of this compound could be more or less required because dimethylsulfide has a characteristic disagreeable odor commonly described as cabbage-like but it is often used in low concentration as additive to confer aromatic flavors to food.

Regarding ketones, diacetyl and acetoin, not detected at time zero, increased during fermentation in all the samples, mainly in those prepared with quinoa flour and fermented with *L. paracasei* I1 (Qpa) and *L. brevis* T4 (Qbr). The occurrence of these compounds is very interesting because acetoin, along with diacetyl, are compounds characterized for a pleasant butter, fatty odors. Because of this, acetoin and diacetyl are often used as a food flavoring in baked goods and as a fragrance. The highest amount of these volatile compounds in the quinoa-based doughs may be correlated to the free amino acids occurred in the doughs during fermentation. Other researchers evidenced that sourdough fermentation with lactic acid bacteria increases proteolysis and subsequently the amino acid concentration, contributing to the improve bread flavor (Hansen et al., 1989; Thiele et al., 2002). This might indicate that the use of alternative flours, rich in proteins, as quinoa and fermented with specific starter can lead to high aminoacids production favoring the formation of specific aroma compounds. Furthermore, *L. paracasei* I1, compared to the other strains, produced high amount of 2,6-dimethyl-4-heptanone, 5-methyl-3-hexanone, 4-methyl-3-penten-2-one, volatile compounds highly appreciated in the food industry for their fruity notes. For this reason *L. paracasei* I1 may be suitable as culture adjunct starter in the bakery industry.

In quinoa fermented dough, moreover, were recorded other ketones compounds (3-octanone, 2-octanone, 1-octen-3-one and 3-octen-2-one), characterized as having a herbal, woody and mushroom odor.

Among esters and acetates, the ethyl acetate was not detectable at time zero but its value increased mainly in the doughs fermented by *L. brevis* T4 (Wbr, Kbr, WQKbr) evidencing the best ability of *L. brevis* species to produce this metabolite compared to *L. paracasei* I1 and *L. plantarum* M4.

Several alcohols was recorded during fermentation. The production of some compounds as 2-furanmethanol, benzyl alcohol and 2-phenylethanol is very interesting because they are featured by a pleasant floral and sweet note. Other authors (Hansen et al., 1989; Rehman et al., 2006), studying sourdough rye bread crumb suggested that a most intense and bread-like flavor was related to propanone, 3-methylbutanal, benzyl alcohol and 2-phenylethanol.

In the doughs produced with Kamut® flour, among furans, prevailed 2-ethylfuran and 2-pentylfuran. Furans are derivatives of furan and are commonly found in heat-treated commercial foods formed by thermal degradation of natural food constituents (European Food Safety Authority, 2011).

Pyrazines were found only in the doughs prepared with quinoa flour (Q and WQK) both at time zero and after fermentation. These compounds are generally characterized by nutty, roasted, cocoa, musty aroma that represent key sensory properties in the food industry. Flavor profiles of pyrazines range from the savory to the sweet. These flavors are used in many application, but especially in foods that need a savory touch. The production of pyrazine is often the product of Maillard-reaction and Strecker degradation. As asserted Pico et al. (in press), pyrazines result from the reaction between Maillard reaction products and lipid oxidation products (Eskin and Shahidi, 2012) that could have taken place during the processing of grain, since the germ of the grain contains a sufficient amount of lipids and there is heat in the medium to carry out Maillard reactions. The presence of high amount of pyrazines in our quinoa dough samples could be dependent on the process to which quinoa seeds were subjected. In fact, to remove from quinoa saponins, cause of bitter taste, usually the seeds are washed. After that, in order to remove the water to a level at which microbial spoilage is minimized, quinoa seeds are dried at temperature between 40 and 70°C for time ranging from 2 to 7 h depending on temperature used (Taylor and Parker, 2002). This drying process could determine the production of furans and pyrazines.

Among volatile acids detected by SPME, the main compounds found were acetic, hexanoic, 2-methylbutanoic and pentanoic acid. The presence of high amount of acetic and isovaleric acid (3-methylbutanoic acid) as such as, pentanoic acid is very important because, as also reported by other authors (Czerny and Schieberle, 2002), they are typical flavor active compounds formed during sourdough fermentation.

In our research, as expected, the volatile compounds occurred in the flours were of minor importance in quality and quantity respect to the fermented doughs. Wheat and Kamut® doughs at time zero presented a comparable but weak aroma, whereas volatile organic composition of quinoa dough just formed was more distinct because quinoa itself represents a highly specific source of volatile organic compounds. During lactic acid bacteria fermentation, instead, the effect of metabolic activities on volatile compounds production was notably noteworthy in both Kamut® and quinoa doughs. The PCA analysis of the 49 volatile compounds recorded after 48 h fermentation allow to better understand the differences between the dough samples. As determined by Principle component analysis (PCA), the quinoa and Kamut® doughs were located in different zones of the plane respect to the wheat dough evidencing the formation of a distinctive heterogeneous aromatic profile in each dough. Quinoa fermented doughs were characterized for specific nutty, roasted and buttery tones, whereas Kamut® doughs were characterized for fruity, rose, green and sweet tones. The supplementation of these flours to wheat flours could allow to develop a nutritionally valuable product with excellent and distinctive sensory acceptance. Moreover, the aromatic profiles of the fermented doughs were greatly differentiated each other indicating that the fermentation add more complexity to the sensory profiles. One of the most interesting strain was *L. paracasei* I1 that produced, compared to *L. brevis* T4 and *L. plantarum* M4, the highest amount of different ketons such as, diacetyl, acetoin, 2,6-dimethyl-4-heptanone, 5-methyl-3-hexanone, 4-methyl-3-penten-2-one, volatile compounds highly appreciated in the bakery products for their buttery, fatty and fruity notes. Furthermore, in our results, quinoa and Kamut® represent an optimal substrate for the growth of lactic acid bacteria. All the strains used, belonging to the species *L. paracasei, L. plantarum* and *L. brevis*, were able to grow in the different substrates although the best microbial growth was recorded in the doughs made with quinoa flour when fermented with *L. paracasei* I2 (10 log CFU/g after 48 h fermentation). A good level of acidification was found in all the fermented doughs even if the highest TTA values and the largest production of volatile organic acids (acetic acid, 2-methylbutanoic acid and hexanoic acid) was recorded in the doughs produced with quinoa flour respect with Kamut® and wheat doughs.

## Conclusion

The key characteristic of bakery products is flavor. Aromatic compounds are primarily generated during baking but are notably influenced by ingredients and fermentation conditions. Usually flours have distinct aromatic characteristics, but they need to undergo several changes in order to produce the characteristic flavor of bread. Prerequisites of formation of the desired bread flavor compounds is the dough fermentation that depends strongly from type of flours and microbial starter used.

In conclusion, a selection of strain starter and substrate combinations may improve simultaneously the sensorial and the health promoting characteristics of cereal-fermented foods. *L. paracasei* may be considered a suitable culture adjunct in the bakery industry and the use of quinoa or Kamut® fermented dough could be interesting for producing more balanced foods with respect to nutritional aspects and characterized for a distinctive volatile compounds.

## Author contributions

AR and TD designed the study, conceived the experiment design; analyzed and interpreted the data and drafted the manuscript. AR, TD, MM, and FB performed the experiments, acquired and interpreted the data and performed the statistical analyses. All authors provided critical revisions and approved the final version of the manuscript.

### Conflict of interest statement

The authors declare that the research was conducted in the absence of any commercial or financial relationships that could be construed as a potential conflict of interest.
